# Irrigation-dependent acute ganglionated plexus modulation during variable-loop pulsed field ablation

**DOI:** 10.1093/europace/euag218

**Published:** 2026-08-21

**Authors:** Masanaru Sawada, Koichi Nagashima, Ryuta Watanabe, Yuji Saito, Yuji Wakamatsu, Naoto Otsuka, Shu Hirata, Moyuru Hirata, Hikaru Masuda, Akinori Matsushima, Masato Sakaki, Shiro Nakahara, Hirotsugu Sato, Hideyuki Aoki, Yuta Kimura, Hidehira Fukaya, Masahide Harada, Yuji Motoike, Mitsuru Takami, Yasuo Okumura

**Affiliations:** Division of Cardiology, Department of Medicine, Nihon University School of Medicine, 30-1 Ohyaguchi-kamicho, Itabashi-ku, Tokyo 173-8610, Japan; Division of Cardiology, Department of Medicine, Nihon University School of Medicine, 30-1 Ohyaguchi-kamicho, Itabashi-ku, Tokyo 173-8610, Japan; Division of Cardiology, Department of Medicine, Nihon University School of Medicine, 30-1 Ohyaguchi-kamicho, Itabashi-ku, Tokyo 173-8610, Japan; Division of Cardiology, Department of Medicine, Nihon University School of Medicine, 30-1 Ohyaguchi-kamicho, Itabashi-ku, Tokyo 173-8610, Japan; Department of Cardiology, Pulmonology, and Nephrology, Yamagata University School of Medicine, Yamagata, Japan; Division of Cardiology, Department of Medicine, Nihon University School of Medicine, 30-1 Ohyaguchi-kamicho, Itabashi-ku, Tokyo 173-8610, Japan; Division of Cardiology, Department of Medicine, Nihon University School of Medicine, 30-1 Ohyaguchi-kamicho, Itabashi-ku, Tokyo 173-8610, Japan; Division of Cardiology, Department of Medicine, Nihon University School of Medicine, 30-1 Ohyaguchi-kamicho, Itabashi-ku, Tokyo 173-8610, Japan; Division of Cardiology, Department of Medicine, Nihon University School of Medicine, 30-1 Ohyaguchi-kamicho, Itabashi-ku, Tokyo 173-8610, Japan; Division of Cardiology, Department of Medicine, Nihon University School of Medicine, 30-1 Ohyaguchi-kamicho, Itabashi-ku, Tokyo 173-8610, Japan; Division of Cardiology, Department of Medicine, Nihon University School of Medicine, 30-1 Ohyaguchi-kamicho, Itabashi-ku, Tokyo 173-8610, Japan; Division of Cardiology, Department of Medicine, Nihon University School of Medicine, 30-1 Ohyaguchi-kamicho, Itabashi-ku, Tokyo 173-8610, Japan; Department of Cardiology, Dokkyo Medical University, Saitama Medical Center, Saitama, Japan; Department of Cardiology, Dokkyo Medical University, Saitama Medical Center, Saitama, Japan; Department of Cardiology, Dokkyo Medical University, Saitama Medical Center, Saitama, Japan; Department of Cardiology, Dokkyo Medical University, Saitama Medical Center, Saitama, Japan; Department of Cardiovascular Medicine, Kitasato University School of Medicine, Sagamihara, Japan; Department of Cardiology, Fujita Health University, Aichi, Japan; Department of Cardiology, Fujita Health University, Aichi, Japan; Section of Arrhythmia, Division of Cardiovascular Medicine, Department of Internal Medicine, Kobe University Graduate School of Medicine, Kobe, Japan; Division of Cardiology, Department of Medicine, Nihon University School of Medicine, 30-1 Ohyaguchi-kamicho, Itabashi-ku, Tokyo 173-8610, Japan

**Keywords:** Pulsed field ablation, Atrial fibrillation, Ganglionated plexus, Heart rate variability, Electroporation, Joule heating

## Abstract

**Clinical trial registration**: UMIN Clinical Trials Registry, UMIN000057894.

Pulsed field ablation (PFA) is considered relatively neural-sparing because electroporation preferentially affects myocardium.^1^ However, biological effects may differ across PFA platforms and procedural settings.^2,3^ Variable-loop circular catheter (VLCC)-based PFA can generate transient Joule heating at the catheter-tissue interface, which is reduced by increased irrigation.^4^ Whether irrigation flow influences acute ganglionated plexus (GP) modulation in humans remains unclear. We therefore compared immediate post-ablation GP responses after radiofrequency ablation (RFA) and VLCC-based PFA and assessed differences between low-flow PFA at 4 mL/min and high-flow PFA at 30 mL/min.

This prospective multicentre observational study (five institutions) included 55 patients undergoing first-time atrial fibrillation ablation with VLCC-based PFA (*n* = 29) or RFA (*n* = 26) using previously described PFA and RFA techniques.^5–7^ The study was approved by the institutional review boards of all participating centres, and all patients provided written informed consent. Within the PFA cohort, 17 patients underwent low-flow PFA using 4 mL/min irrigation and 12 underwent high-flow PFA using 30 mL/min irrigation. Study flow is shown in *Figure 1A*. GP function was assessed using high-frequency stimulation (HFS; 20 Hz, 25 mA, 10-ms pulse width, 5 s) at five major left atrial GP regions. Acute GP response loss was defined as disappearance of the previously inducible HFS-positive response.^8^ Only baseline-positive sites were reassessed immediately after completion of pulmonary vein isolation (PVI), before cavotricuspid isthmus or superior vena cava isolation when these were performed, and without a predefined waiting period. Tissue proximity indication was used during PFA,^5,6^ and intracardiac echocardiography or multipolar mapping was used according to institutional workflow. Biomarkers were obtained at baseline and after ablation, including Day 1, defined as the morning after the procedure. Fourteen-day ambulatory ECG monitoring (Fukuda Denshi Co., Ltd., Tokyo, Japan) was performed at baseline and at 1 and 3 months. Heart rate variability (HRV), including the standard deviation of normal-to-normal intervals (SDNN), was analysed only from recordings with evaluable sinus-rhythm data; periods of atrial fibrillation or atrial tachycardia were excluded. Patient-clustered logistic regression with generalized estimating equations (GEE) was used for site-level GP outcomes.

**Figure 1:**
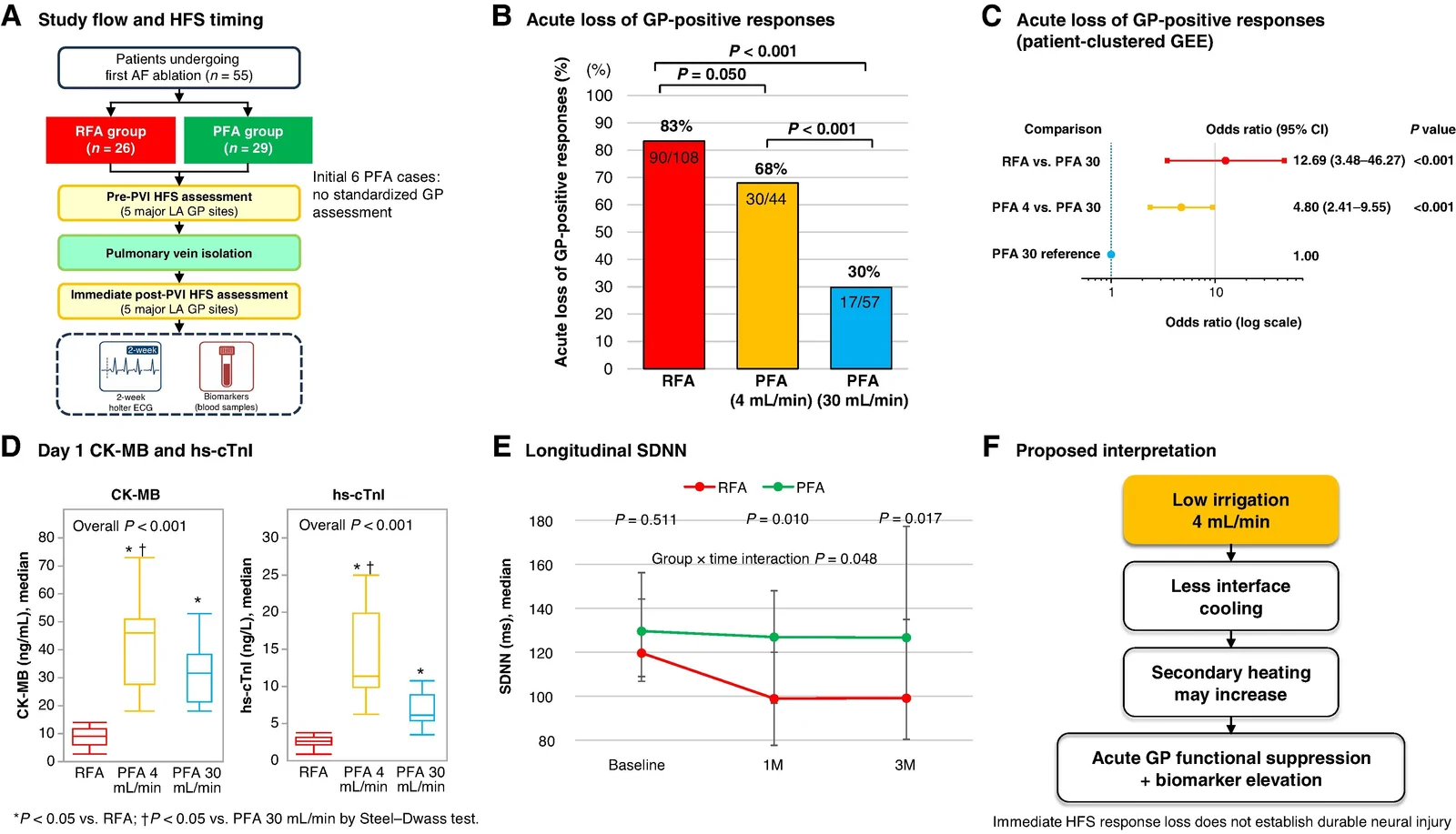
Irrigation-dependent acute ganglionated plexus modulation during variable-loop pulsed field ablation. *(A)* Study flow and timing of HFS reassessment. The initial six PFA cases were enrolled before implementation of the standardized GP mapping protocol and therefore did not undergo GP assessment. Post-ablation HFS was performed immediately after PVI and before CTI or SVC isolation when these were performed. *(B)* Acute loss of GP-positive responses after RFA, low-flow PFA (4 mL/min), and high-flow PFA (30 mL/min). *(C)* Patient-clustered GEE analysis for acute GP response loss, using high-flow PFA as the reference. *(D)* Day 1 CK-MB and hs-cTnI levels according to ablation modality and irrigation flow. Both biomarkers were significantly higher after low-flow than after high-flow PFA. *(E)* Longitudinal SDNN derived from evaluable sinus-rhythm data on 14-day ambulatory ECG monitoring in the RFA and pooled PFA groups. *(F)* Proposed interpretation: low-flow irrigation may increase secondary heating at the catheter–tissue interface, contributing to acute functional GP suppression and biomarker release; however, immediate HFS response loss does not establish durable neural injury. CK-MB, creatine kinase-MB; CTI, cavotricuspid isthmus; GEE, generalized estimating equations; GP, ganglionated plexus; HFS, high-frequency stimulation; hs-cTnI, high-sensitivity cardiac troponin I; PFA, pulsed field ablation; PVI, pulmonary vein isolation; RFA, radiofrequency ablation; SDNN, standard deviation of NN intervals; SVC, superior vena cava; VLCC, variable-loop circular catheter.

A total of 209 baseline HFS-positive GP responses were identified. Acute GP response loss was more frequent after RFA than after PFA (83% vs. 47%, *P* < 0.001). Within the PFA group, response loss was greater with low-flow than with high-flow PFA (68% vs. 30%, *P* < 0.001; *Figure 1B*). In patient-clustered logistic regression using GEE and adjusted for CHA_2_DS_2_-VASc score, RFA and low-flow PFA were associated with greater acute GP response loss than high-flow PFA (adjusted odds ratios 12.69, 95% confidence interval 3.48–46.27, and 4.80, 95% confidence interval 2.41–9.55, respectively; both *P* < 0.001; *Figure 1C*). Day 1 myocardial injury biomarkers differed significantly according to ablation modality and irrigation flow. Both creatine kinase-MB (CK-MB) and high-sensitivity cardiac troponin I (hs-cTnI) were significantly higher after low-flow than after high-flow PFA (*Figure 1D*). Among patients with evaluable sinus-rhythm data on 14-day ambulatory ECG monitoring, SDNN was lower after RFA than after PFA at 1 and 3 months (*Figure 1E*), suggesting more persistent autonomic remodelling after RFA. No major procedural complications occurred.

Three findings are noteworthy. First, VLCC-based PFA produced less immediate GP response loss than RFA. Second, the acute GP effect of PFA was strongly irrigation-dependent despite use of the same catheter platform and pulse sequence. Third, low-flow PFA was accompanied by greater CK-MB and hs-cTnI release, whereas longer-term autonomic remodelling remained less pronounced than after RFA. These findings are consistent with, but do not prove, a contribution of irrigation-sensitive secondary heating. Increased irrigation may reduce catheter-tissue interface temperature rise and thereby attenuate acute functional effects on adjacent GP tissue (*Figure 1F*). However, tissue temperature was not measured directly, and differences in lesion formation, catheter contact, or regional GP distribution may also have contributed. Although the treatment groups differed in age, CHA_2_DS_2_-VASc score, and anaesthetic strategy, baseline resting heart rate and HRV indices were broadly comparable. Because HFS was repeated immediately after PVI, the endpoint should be interpreted as acute functional GP suppression rather than durable denervation. The dissociation between immediate HFS response loss and relatively preserved HRV after PFA suggests that at least part of the acute effect may be reversible. This interpretation is consistent with prior reports showing limited durable autonomic change after PFA despite intraprocedural vagal responses or transient GP modulation.^9,10^ These findings further suggest that irrigation settings may influence not only myocardial lesion characteristics but also acute biological effects on adjacent neural structures.

This study was observational, non-randomized, and small. Treatment modality, irrigation flow, and anaesthetic strategy were not randomized, and residual confounding remained despite patient-clustered analysis. Multiple HFS observations were obtained within each patient; therefore, simple site-level proportions are descriptive. Site-specific GP estimates were not emphasized because baseline GP positivity varied by anatomical region and the number of responses at individual sites was small. Contact assessment and imaging workflows varied among centres. HRV analyses were restricted to evaluable sinus-rhythm recordings and may have been influenced by medication use and other physiological factors. Intracranial microembolic signals were not monitored using transcranial Doppler, and brain magnetic resonance imaging was not systematically performed. Therefore, we could not determine whether lower irrigation was associated with gaseous or thromboembolic cerebral events, despite the absence of clinically apparent stroke or transient ischaemic attack. Tissue temperature was not measured, making the proposed thermal contribution inferential. Findings apply specifically to the VLCC platform and pulse sequence studied and may not apply to other PFA systems or to updated waveforms designed to reduce temperature rise.

## Conclusions

VLCC-based PFA produced less acute GP response loss than RFA, but its effect was strongly irrigation-dependent. Greater acute GP suppression and higher CK-MB and hs-cTnI release with low-flow PFA are consistent with a contribution of irrigation-sensitive secondary heating. Because GP reassessment was performed immediately after PVI, these findings indicate acute functional modulation and do not establish durable neural injury.

## Data Availability

The data underlying this article are available from the corresponding author upon reasonable request but are not publicly available due to privacy and institutional restrictions.
